# A retrospective analysis of the effect of latent tuberculosis infection on clinical pregnancy outcomes of *in vitro* fertilization–fresh embryo transferred in infertile women

**DOI:** 10.1515/med-2023-0870

**Published:** 2023-12-06

**Authors:** Xinzhuan Jia, Lan Wei, Na Zhang, Bolin Zheng, Mengya Li, Hongxia Wang, Erhuan Liu, Jie Xu, Guangyu Sun, Zhengmao Zhang

**Affiliations:** Department of Reproductive Medicine, The Fourth Hospital of Hebei Medical University, Shijiazhuang 050041, Hebei Province, China; Hebei Chest Hospital, Shijiazhuang 050041, Hebei Province, China; Department of Gynaecology, The Fourth Hospital of Hebei Medical University, Shijiazhuang 050041, Hebei Province, China; Shenzhou People’s Hospital, Shenzhou, 053800, Hebei Province, China; Department of Gynaecology, The Fourth Hospital of Hebei Medical University, No. 12, Jiankang Road, Shijiazhuang 050041, Hebei Province, China

**Keywords:** latent tuberculosis infection, fresh embryo transfer, *in vitro* fertilization, pregnancy outcome

## Abstract

In areas with high incidence of tuberculosis (TB), there are more infertile women who underwent *in vitro* fertilization (IVF) and have latent TB infection (LTBI), and thus, their potential risks should be paid enough attention. The purpose of our study aimed to analyze the relationship between LTBI and clinical pregnancy outcomes of IVF and fresh embryo transfer (IVF–FET). This was a retrospective study of 628 infertile women who had undergone IVF–FET in the Fourth Affiliated Hospital of Hebei Medical University from January 2019 to December 2021. The women experienced no clinical symptoms, negative imaging, and T-SPOT.TB-positive diagnosis of LTBI. We divided the study population into the LTBI group and the non-LTBI group. The clinical pregnancy rate in the LTBI group was significantly lower than that in the non-LTBI group (40.54% vs 49.51%, *P* = 0.031), and there was no significant difference in live birth rate and miscarriage rate between the two groups. Logistic regression analysis showed that LTBI was an independent risk factor for decreased clinical pregnancy rate in infertile women undergoing IVF–FET. In conclusion, LTBI affects clinical pregnancy rate of IVF–FET in infertile women, and therefore, clinicians (especially in countries with a high TB burden) need to pay attention to LTBI before IVF and embryo transfer.

## 1 Introduction

Tuberculosis (TB) is a public health and social problem of global concern. In 2020, an estimated 10 million new TB cases were reported globally, with adult women accounting for 32%. Two-thirds of the new cases were in eight developing countries, with China accounting for 8.4% of the total reported cases [1]. Among new cases, about 80% of TB patients were converted from latent TB infection (LTBI) [2].

TB is one of the main causes of infertility [3]. It is estimated that about 5–13% of reproductive women aged between 20 and 40 years have LTBI [4]. With the popularization of assisted reproductive technology, more and more infertile patients are undergoing *in vitro* fertilization and embryo transfer (IVF–ET) to become pregnant. In areas with high incidence of TB, such as China and India, the number of women undergoing IVF should be higher and the potential risks should be taken seriously. For high-risk groups, timely detection of LTBI and preventive anti-TB treatment (ATT) are important measures for TB prevention and control. There are currently no published studies analyzing whether LTBI affects clinical pregnancy outcomes of IVF–ET. To assess the relationship between LTBI and clinical pregnancy outcomes of IVF and fresh embryo transfer (IVF–FET), we conducted the retrospective study based on a case-registry database.

## 2 Materials and methods

### 2.1 Research design

This is a retrospective study, whose subjects were infertile women who underwent IVF–FET at the Department of Reproductive Medicine of the Fourth Affiliated Hospital of Hebei Medical University from January 1, 2019, to December 31, 2021. The inclusion criteria were: (1) age of infertile women ≥18 years; (2) detecting T-SPOT.TB before IVF–ET treatment; (3) there was no abnormality in chest CT before IVF–FET treatment; (4) fresh embryo transfer; and (5) complete medical records. The exclusion criteria were: (1) previous history of TB and ATT; (2) active pulmonary TB; (3) diabetes mellitus, malignant tumors, AIDS, and autoimmune diseases; (4) receiving glucocorticoid or/and immunosuppressant therapy within the last 3 months; (5) chromosomal abnormalities; and (6) IVF–ET caused by male factors. The data needed for this study came from the case registration database of the Fourth Affiliated Hospital of Hebei Medical University.

A total of 1,131 cases were screened according to the inclusion criteria. According to exclusion criteria, 503 cases were excluded. Of the remaining 628 patients, there were 222 LTBI cases and 406 non-LTBI cases. A total of 481 patients (76.59%) [including 176 patients (79.28%) in the LTBI group and 305 patients (75.12%) in the non-LTBI group] had undergone laparoscopy/hysteroscopy before IVF–FET and were histologically negative (TB-DNA, NTM-DNA). The other patients did not undergo laparoscopy/hysteroscopy because the salpingography and pelvic B-ultrasound results were normal.

This retrospective analysis was conducted in accordance with the World Medical Association Code of Ethics (Declaration of Helsinki) and was approved by the Ethics Committee of the Fourth Affiliated Hospital of Hebei Medical University (approval number: 2021KS010). All the patients gave up the need for informed consent.

### 2.2 T-SPOT.TB detection

T-SPOT.TB of the peripheral blood was tested within 3 months prior to IVF treatment. The T-SPOT.TB test kit is manufactured by Oxford Immunote, UK. Five milliliters of peripheral venous blood of every subject was mixed into a heparin-anticoagulated test tube immediately after the collection, and the peripheral blood mononuclear cells were isolated within 4 h to prepare the cell suspension. The collected suspension was transferred to an antigen-coated four-well plate. In these wells, positive (phytohemagglutinin, PHA) and negative controls, Panel A (early secretory antigen targe-6, ESAT-6) and Panel B (culture filtrate proteins, CFP-10), were analyzed. Then, the cell suspension of 100 μl containing 2.5 × 10^5^ cells was added into every single well. The prepared plate was incubated for 16–20 h in a 5% CO_2_ oven at 37°C. The enzyme-linked immunospot assay (ELISPOT) reader (CTL-ImmunoSpotS5 Versa Analyser) was used to read spot-forming cells (SFCs). According to the reaction of antigen A or antigen B hole, when the SFC in the negative control hole equals 0–5, (antigen A or antigen B hole SFC) − (negative control hole SFC) ≥ 6, the result is positive; when the SFC in the negative control hole equals 6 to10, (antigen A or antigen B hole SFC) ≥ 2 × (negative control hole SFC), the result is also positive. Otherwise, it is negative.

### 2.3 Research objects grouping

**Figure 1:**
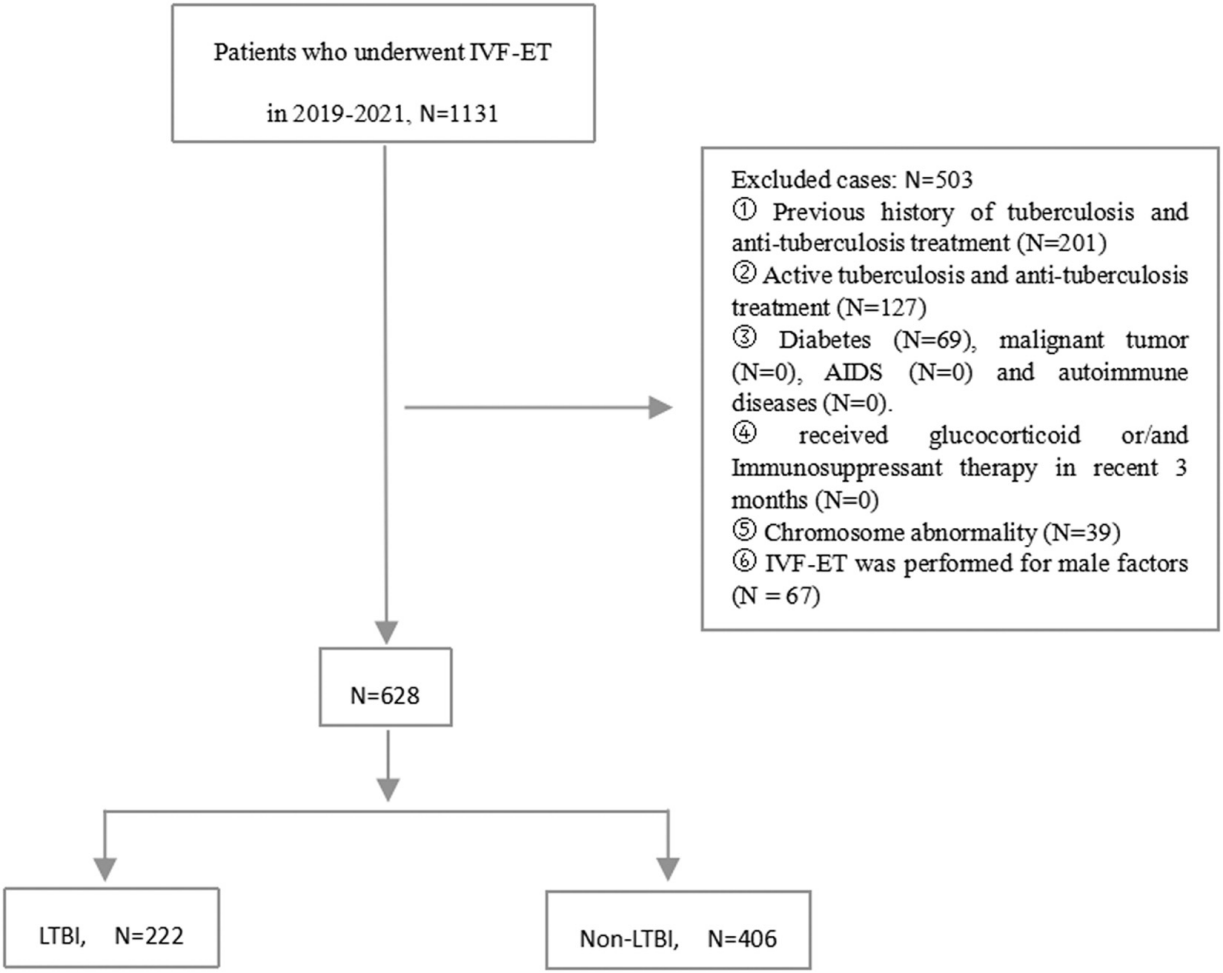
Study flow chart.

According to the results of peripheral blood interferon-γ release assay (IGRA) before IVF treatment, the subjects were divided into two groups: no TB-related symptoms and no evidence of active TB in bacteriology and imaging examinations. T-SPOT.TB positive was defined as the LTBI group while T-SPOT.TB negative was defined as the non-LTBI group (Figure 1).

### 2.4 IVF–ET scheme

All the subjects had undergone the cycle of ovarian stimulation, egg retrieval, and IVF–FET. Ovarian stimulation regiments include gonadotropin-releasing hormone agonist (GnRH-a) long regimen, GnRH-a ultra-long regimen, and GnRH antagonist regimen. The choice was based on the doctor’s discretion. Ultrasound monitoring was conducted and serum estradiol (E_2_) levels were measured during ovulation induction. Human chorionic gonadotropin (hCG) triggered ovulation when the dominant follicle diameter was ≥18 mm and E_2_ levels reached 150–300 ng/L. About 36 h after hCG administration, cumulus–oocyte complexes were recovered under ultrasound guidance, and IVF was performed. On the third day, the embryos in the cleavage stage were scored and graded (based on blastomere number, cell symmetry, and division). I–III cleavage stage embryos were identified as transferable embryos. Phase I–II embryos were defined as good-quality embryos which met the following criteria: (1) containing 6–8 cells 3 days after fertilization; (2) containing homogeneous blastomere; and (3) the degree of breakage was less than 10%. On the same day, these fresh embryos were transferred to the patients.

### 2.5 Evaluation indicators

Clinical pregnancy rate = Number of clinical pregnancies/Number of transplanted embryos × 100% (1)

Live birth rate = Number of live births/Number of embryos transferred × 100%, (2)

Miscarriage rate = Number of miscarriages/Number of clinical pregnancies × 100% (3)

### 2.6 Pregnancy outcome

The primary outcome is the clinical pregnancy rate. Clinical pregnancy is defined by embryo sac detection through ultrasound 30 days after embryo transfer. Secondary outcomes are live birth rate and miscarriage rate. Live birth is defined as the delivery of a viable infant. Miscarriage is defined as fetus loss before the 24th week of pregnancy [5].

### 2.7 Statistical analysis

Statistical analysis was performed using SPSS 20.0. Continuous variables were expressed as mean ± standard deviation (SD) or as median [interquartile range (IQR)] and were analyzed using independent-sample *t*-tests or the Mann–Whitney *U* test, as appropriate. Categorical variables were summarized with numbers (*N*) and proportions (%), and were analyzed using the chi-square test. Chi-square test was used to compare the pregnancy outcomes between the LTBI group and the non-LTBI group. Multivariate logistic regression was used to assess the effects of exposure factors on the pregnancy outcomes. Two-sided *P*-value <0.05 was considered statistically significant.

## 3 Result

### 3.1 Baseline data of infertile patients in the LTBI and non-LTBI groups

The LTBI and non-LTBI groups were compared for age, body mass index (BMI), duration of infertility, causes of infertility (unknown reasons, fallopian tube factors, other female factors), COH regimen (GnRH-a long regimen, GnRH-a ultra-long regimen, GnRH antagonist regimen), basal hormone levels (follicle-stimulating hormone, luteinization hormone, E_2_, testosterone), anti-Müllerian hormone (AMH), number of total sinus follicles, endometrium thickness on hCG day, and number of good-quality embryos on the third day, and there was no significant difference (*P* > 0.05, Table 1).

**Table 1:** Characteristics of infertile patients undergoing fresh embryo transfer

Characteristics	LTBI group (*N* = 222)	Non-LTBI group (*N* = 406)	*t*/*U*/*χ* ^2^	*P* value
Age, years (mean ± SD)	32.72 ± 5.05	32.46 ± 5.56	0.573	0.567
BMI, kg/m^2^ (mean ± SD)	22.05 ± 1.83	22.28 ± 2.07	1.392	0.165
Duration of infertility, years [median (IQR)]	3.00 (2.00, 5.00)	4.00 (2.00, 5.00)	450.86	0.992
Causes of infertility, *N* (%)				
Fallopian tube factors	139 (62.61%)	253 (62.31%)	0.328	0.849
Unknown reasons	39 (17.57%)	66 (16.26%)		
Other female factors	44 (19.82%)	87 (21.43%)		
Baseline hormone (mean ± SD)				
FSH, mIU/mL	6.60 ± 1.36	6.73 ± 1.36	1.206	0.228
LH, mIU/mL	6.31 ± 1.73	6.15 ± 1.41	1.158	0.247
E_2_, pg/mL	42.35 ± 6.22	42.15 ± 5.29	0.394	0.694
T, ng/mL	0.24 ± 0.13	0.22 ± 0.12	1.620	0.106
AMH, ng/mL (mean ± SD)	3.84 ± 1.72	3.98 ± 1.88	0.928	0.354
COH regimen, *N* (%)				
GnRH-a long	122 (54.95%)	225 (55.42%)	0.526	0.769
GnRH-a ultra-long	19 (8.56%)	41 (10.10%)		
GnRH antagonist	81 (36.49%)	140 (34.48%)		
Endometrial thickness (hCG day), mm (mean ± SD)	10.31 ± 1.86	10.51 ± 1.71	1.295	0.196
No. of total sinus follicles (mean ± SD)	16.45 ± 6.79	17.12 ± 8.59	0.689	0.401
No. of good-quality embryos (Day 3) [median (IQR)]	2.00 (1.00, 3.00)	2.00 (1.00, 3.00)	448.43	0.917

BMI: body mass index; FSH: follicle-stimulating hormone; LH: luteinizing hormone; E_2_: estradiol; T: testosterone; AMH: anti-Müllerian hormone; COH: controlled ovarian hyperstimulation; GnRH: gonadotropin-releasing hormone; hCG: human chorionic gonadotropin.

### 3.2 Pregnancy outcomes in the LTBI and non-LTBI groups

The clinical pregnancy rate in the LTBI group was lower than that in the non-LTBI group (*P* < 0.05). Compared with the non-LTBI group, the LTBI group had a lower live birth rate and higher miscarriage rate, but there was no statistical significance (*P* > 0.05, Table 2).

**Table 2:** Pregnancy outcomes in the LTBI and non-LTBI groups

Pregnancy outcomes	LTBI group (*N* = 222)	Non-LTBI group (*N* = 406)	*χ* ^2^	*P* value
Clinical pregnancy rate	40.54% (90/222)	49.51% (201/406)	4.641	0.031
Miscarriage rate	21.11% (19/90)	20.40% (41/201)	0.019	0.889
Live birth rate	25.23% (56/222)	30.54% (124/406)	1.984	0.159

### 3.3 Factors associated with pregnancy outcomes in infertile patients of IVF–ET

Multivariate logistic regression showed that LTBI (odds ratio [OR] = 0.695, 95% confidence interval [CI] 0.499–0.968, *P* = 0.032), age (OR = 0.953, 95% CI 0.935–0.982, *P* = 0.002), AMH (OR = 1.133，95% CI 1.038–1.236, *P* = 0.005), the number of good-quality embryos (OR = 1.139, 95% CI 1.004–1.243, *P* = 0.003), and COH regimen (*P* = 0.016) have significant effects on clinical pregnancy outcomes. After correcting these indicators, we found that LTBI was an independent risk factor for the decrease of clinical pregnancy rate in infertile women undergoing IVF–FET (OR 0.694, 95% CI 0.494–0.975, *P* = 0.035). LTBI (OR = 0.833, 95% CI 0.471–1.474, *P* = 0.531) had no significant effect on miscarriage. LTBI (OR = 0.767, 95% CI 0.530–1.110, *P* = 0.160) had no significant effect on live births.

## 4 Discussion

Female genital TB (FGTB) is not an uncommon cause of infertility worldwide, especially in developing countries. IVF–ET has been shown to improve pregnancy outcomes in infertile women with FGTB. There is limited evidence on the clinical outcomes of IVF–ET in LTBI patients. In our study, a total of 628 patients were involved. The clinical pregnancy rate in the LTBI group was significantly lower than that in the non-LTBI group, and after correcting relevant indicators, LTBI was found to be an independent risk factor for the decrease in clinical pregnancy rate in the infertile women undergoing IVF–FET.

Currently, there is no gold standard for diagnosing LTBI. At present, the IGRA series products widely used in the world mainly include Quantiferon-TB gold In-tube (QFT-GIT) based on enzyme-linked immunosorbent assay and T-SPOT.TB based on ELISPOT. The sensitivity of QFT-GIT is largely affected by the immune status of the host, while the sensitivity of T-SPOT.TB is mainly affected by the antigen load. Our study was grouped according to the results of T-SPOT.TB. The T-SPOT.TB-positive subjects were included in the LTBI groups, of which 79.28% underwent laparoscopy/hysteroscopy without evidence of active genital TB infection and 20.72% did not undergo laparoscopy/hysteroscopy due to normal results of salpingography and pelvic B-ultrasonography. Subjects with active TB were not allowed to undergo IVF–ET.

Some scholars have reported that clinical pregnancy rate and live birth rate of IVF in TB patients, who have not previously received ATT, are lower than that of those who have previously received ATT, especially those with unexplained infertility [6]. The implantation rate and pregnancy rate of IVF in patients with tubal TB are similar to that in those without TB. However, compared to patients with tubal TB or without TB, those with endometrial TB experience significantly lower fertilization rate, good-quality embryo rate, and implantation rate, resulting in a decrease in the cumulative pregnancy rate [7]. Lin et al. reported that cured endometrial TB is associated with low live birth rates [8]. Different from our findings, this can be explained by the fact that the cohort and subgroup analyses vary across the studies.

Immunity plays an important role in both latent and active TB [9]. The three factors affecting successful implantation and pregnancy are uterine receptivity, endometrial regeneration, and cytokine regulation. LGTB-associated immune factors (such as vascular endothelial growth factor, various cytokines, leukemia inhibitors, and cell adhesion molecules such as E cadherin, mucin-1, MECA-79, and alphavbeta3 integrin) may play a role in the implantation of the zygote by changing endometrial receptivity [10,11,12,13,14,15,16]. Highly activated immune disorders of T-receptor cells and CD4+ lymphocytes have also been observed in LGTB, leading to implantation failure [17,18]. On the other hand, progesterone used during IVF may lead to increased immunosuppression of the Th1 response and decreased CD4+ T-cell proliferation [19], and TB may amplify this side effect into a clinical phenomenon. Undergoing IVF treatment may make women more susceptible to TB infection and be associated with worse perinatal outcomes [20].

However, there are still some limitations concerning this study. First, it was a single-center retrospective analysis with a small sample size. Second, the data did not include TB activity and new infections undergoing IVF treatment. In addition, multivariate logistic analysis was used in our study and the results are still indicative and need to be validated in a larger cohort.

## 5 Conclusion

LTBI is associated with lower clinical pregnancy rates in women undergoing IVF–FET. Clinicians (especially in high TB burden countries) need to pay attention to LTBI before IVF–ET.

## Supplementary Material

Supplementary Table

Supplementary material
