# Supplementary material for: A retrospective analysis of the effect of latent tuberculosis infection on clinical pregnancy outcomes of in vitro fertilization–fresh embryo transferred in infertile women
Source: Open Med (Wars). 2023 Dec 6;18(1):20230870. doi: 10.1515/med-2023-0870 (PMC10710212; doi:10.1515/med-2023-0870)
Supplement: Supplementary Table [file med-2023-0870-sm.pdf]

Supplementary material

Clinical pregnancy as the dependent variable

	β	S.E.	Wald	df	Sig.	Exp (β)	95%C.I. for Exp (β)	
							Lower	Upper
Group (1)	−0.363	0.169	4.624	1	0.032	0.695	0.499	0.968
Contant	−0.020	0.099	0.039	1	0.843	0.980		

	β	S.E.	Wald	df	Sig.	Exp (β)	95%C.I. for Exp (β)	
							Lower	Upper
Age	−0.048	0.015	10.047	1	0.002	0.953	0.925	0.982
Contant	1.416	0.499	8.058	1	0.005	4.120		

	β	S.E.	Wald	df	Sig.	Exp (β)	95%C.I. for Exp (β)	
							Lower	Upper
BMI	−0.054	0.040	1.778	1	0.182	1.055	0.975	1.143
Contant	−1.346	0.903	2.221	1	0.136	0.260		

	β	S.E.	Wald	df	Sig.	Exp (β)	95%C.I. for Exp (β)	
							Lower	Upper
FSH	−0.090	0.059	2.308	1	0.129	0.914	0.814	1.026
Contant	0.455	0.404	1.270	1	0.260	1.576		

	β	S.E.	Wald	df	Sig.	Exp (β)	95%C.I. for Exp (β)	
							Lower	Upper
E_2	0.008	0.014	0.321	1	0.571	1.008	0.980	1.037
Contant	−0.487	0.606	0.646	1	0.422	0.614		

	β	S.E.	Wald	df	Sig.	Exp (β)	95%C.I. for Exp (β)	
							Lower	Upper
LH	−0.015	0.052	0.082	1	0.774	0.985	0.889	1.092
Contant	−0.054	0.334	0.026	1	0.873	0.948		

	β	S.E.	Wald	df	Sig.	Exp (β)	95%C.I. for Exp (β)	
							Lower	Upper
T	0.037	0.637	0.003	1	0.954	1.037	0.298	3.613
Contant	−0.155	0.167	0.867	1	0.352	0.856		

	β	S.E.	Wald	df	Sig.	Exp (β)	95%C.I. for Exp (β)	
							Lower	Upper
AMH	0.125	0.045	7.785	1	0.005	1.133	1.038	1.236
Contant	−0.638	0.194	10.815	1	0.001	0.529		

	β	S.E.	Wald	df	Sig.	Exp (β)	95%C.I. for EXP (β)	
							Lower	Upper
No.of good quality embryos	0.130	0.044	8.614	1	0.003	1.139	1.044	1.243
Contant	−0.427	0.126	11.539	1	0.001	0.653		

	β	S.E.	Wald	df	Sig.	Exp (β)	95%C.I. for Exp (β)	
							Lower	Upper
COH protocol			8.297	2	0.016			
COH protocol (1)	0.392	0.306	1.645	1	0.200	1.480	0.813	2.696
COH protocol (2)	0.733	0.294	6.218	1	0.013	2.082	1.170	3.706
Contant	−0.693	0.274	6.406	1	0.011	0.500		

	β	S.E.	Wald	df	Sig.	Exp (β)	95%C.I. for Exp (β)	
							Lower	Upper
Group (1)	−0.365	0.174	4.428	1	0.035	0.694	0.494	0.975
Age	−0.046	0.016	8.583	1	0.003	0.955	0.926	0.985
AMH	0.091	0.047	3.808	1	0.051	1.095	1.000	1.200
No.of good quality embryos	0.130	0.046	8.165	1	0.004	1.139	1.042	1.245
COH protocol			7.624	2	0.022			
COH protocol (1)	0.363	0.314	1.337	1	0.248	1.437	0.777	2.659
COH protocol (2)	0.708	0.301	5.516	1	0.019	2.030	1.124	3.665
Contant	0.315	0.621	0.258	1	0.612	1.371		

Miscarriage as the dependent variable

	β	S.E.	Wald	df	Sig.	Exp (β)	95%C.I. for Exp (β)	
							Lower	Upper
Group (1)	−0.182	0.291	0.393	1	0.531	0.833	0.471	1.474
Contant	−2.186	0.165	176.190	1	0.000	0.112		

Live birth as the dependent variable

	β	S.E.	Wald	df	Sig.	Exp (β)	95%C.I. for Exp (β)	
							Lower	Upper
Group (1)	−0.265	0.188	1.979	1	0.160	0.767	0.530	1.110
Contant	−0.822	0.108	58.142	1	0.000	0.440		
